# Organ-Resolved Untargeted LC-MS/MS Profiling of Putative Metabolite Families in Aerial Organs of *Vitex negundo* L.

**DOI:** 10.3390/metabo16090633

**Published:** 2026-08-31

**Authors:** Menghu Wang, Xinyang Wang, Xue Jiang, Yafeng Zuo, Dong Liu, Qianqian Jiang, Xiangsong Meng

**Affiliations:** School of Traditional Chinese Medicine and Health, Bozhou University, Bozhou 236800, China; 2017070004@bzuu.edu.cn (M.W.); 2023070001@bzuu.edu.cn (X.W.); 2023070019@bzuu.edu.cn (X.J.); zuoyafeng@bzuu.edu.cn (Y.Z.); 2025070004@bzuu.edu.cn (D.L.)

**Keywords:** *Vitex negundo*, Verbenaceae, untargeted metabolomics, LC-MS/MS, organ-resolved profiling, medicinal-resource evaluation, putative metabolite families, plant specialized metabolism, putative annotation

## Abstract

Background/Objectives: *Vitex negundo* L. is a multi-part medicinal and aromatic plant, but organ-resolved chemical information for its aerial organs remains incomplete. This study generated an exploratory paired untargeted liquid chromatography–tandem mass spectrometry (LC-MS/MS) dataset for flowers, stems, and leaves and prioritized organ-associated putative metabolite families for subsequent validation. Methods: To reduce inter-individual background variation, flower, stem, and leaf tissues were harvested from the same three *V. negundo* individuals to form *n* = 3 paired plant blocks. LC-MS/MS feature data were filtered using pooled quality-control relative standard deviation (QC RSD ≤ 30%), median-normalized and log2-transformed. Principal component analysis (PCA) provided an unsupervised overview, and paired feature-level comparisons were performed using paired *t*-tests with Benjamini−Hochberg false discovery rate (FDR) correction. Results: A total of 42,125 QC-filtered LC-MS features were retained. PCA showed organ-associated separation among flowers, stems, and leaves. Using FDR < 0.05 and |mean paired log2 difference| ≥ 1, paired screening yielded 13,043, 22,836, and 13,393 FDR-supported LC-MS features for flower vs. stem, flower vs. leaf, and stem vs. leaf comparisons, respectively. Higher-scoring putative annotations suggested organ-associated putative family-level patterns, including flavonoid-related, hydroxycinnamic-acid derivative, phenolamide-related, triterpenoid-related, iridoid-related, and lignan-related signals. Conclusions: This organ-resolved dataset provides a chemical-feature framework for *V. negundo* aerial organs and putative family-level patterns for future structural confirmation, targeted quantification, bioactivity evaluation, and quality-marker development. Feature counts are not equivalent to unique metabolite counts, and compound labels require validation before being interpreted as confirmed structures.

## 1. Introduction

*Vitex negundo* L. (Verbenaceae), commonly known as Chinese chaste tree, five-leaved chaste tree, Nirgundi, or Lagundi, is an aromatic shrub or small tree widely recorded in tropical and subtropical Asia and several other warm regions [1]. In traditional materia medica and regional folk practices, *V. negundo* is not limited to a single medicinal organ; its leaves, roots, seeds, fruits, and aerial parts have been used for inflammation-, pain-, cough-, skin-, and parasite-related conditions [1]. Modern phytochemical and pharmacological reviews describe *V. negundo* as a medicinal aromatic plant containing multiple classes of specialized constituents and showing diverse bioactivities [2]. Leaves are the most frequently investigated organ in modern pharmacological studies, with reported anti-inflammatory and analgesic activities of mature fresh leaves and antinociceptive activity of leaf extracts [3,4]. Extracts or fractions of *V. negundo* have also shown antioxidant, antibacterial, and antifungal activities in different experimental contexts [5,6,7]. These organ- and extract-focused studies provide a pharmacological and phytochemical basis for the medicinal value of *V. negundo*, but they do not sufficiently explain how chemical features are organized among different plant organs.

Reported constituents of *V. negundo* include flavonoids, phenolic acids, iridoids, terpenoid-related constituents, lignans, and volatile components [2]. A flavonoid glycoside isolated from *V. negundo* has been associated with antifungal activity, supporting the relevance of flavonoid-related chemistry in this species [7]. Together with the reported leaf pharmacology and extract bioactivities, these phytochemical findings suggest that the use or investigation of different organs may be associated, at least in part, with organ-dependent chemical composition [3,4,5,6,7]. Previous part-specific studies further support organ-dependent chemical variation: a targeted liquid chromatography–mass spectrometry (LC-MS) study of *V. negundo* var. cannabifolia compared leaves, seeds, and roots and reported part-dependent distributions of phenolic acids, flavonoids, and iridoids [8]; a recent liquid chromatography–tandem mass spectrometry (LC-MS/MS) study characterized antioxidant-related constituents in *V. negundo* leaves [9]; and an ultra-performance liquid chromatography–quadrupole time-of-flight mass spectrometry (UPLC-Q-TOF/MS) comparison of root, stem/leaf, and fruit tissues in *V. negundo* var. cannabifolia reported differences involving flavonoids, lignans, terpenes, phenylpropanoids, and phenolic acids [10]. These studies establish an organ-specific chemical background, but a paired comparison of flowers, stems, and leaves collected from the same individual *V. negundo* plants under one untargeted LC-MS/MS workflow remains limited.

Organ-dependent chemical differentiation is consistent with the tissue-specific nature of plant specialized metabolism. Specialized metabolites are shaped by tissue function, developmental status, and ecological interactions, rather than being distributed uniformly throughout the whole plant [11,12]. Plant chemical diversity is increasingly interpreted as an adaptive and context-dependent metabolic phenotype [13,14]. Leaves are photosynthetic and light-exposed tissues, and flavonoids or other phenolic compounds often contribute to ultraviolet protection, redox regulation, and defense [15,16]. Stems contain vascular and supporting tissues, where phenylpropanoid-derived compounds may be associated with cell-wall-related metabolism, lignification, and defense responses [17,18]. Flowers are reproductive organs and may accumulate specialized metabolites related to pigmentation, volatile emission, pollinator interaction, and reproductive protection [11,19]. These organ functions provide a biological basis for comparing flowers, stems, and leaves of *V. negundo* and for linking multi-part medicinal use with organ-level chemical differentiation.

An organ-resolved comparison is also relevant to medicinal-resource evaluation. If flowers, stems, and leaves contain different metabolite-family profiles, these differences may help explain why some organs have been preferentially used or studied and may identify less-studied organs that merit further targeted evaluation. Such information is important for quality-control marker selection, resource-organ assessment, and the design of follow-up pharmacological studies [20,21]. Nevertheless, the available chemical evidence was generated across different tissue sets, analytical objectives, and botanical materials. A paired flower-stem-leaf chemical-feature framework can therefore provide a more direct within-plant assessment of aerial-organ differences.

Mass-spectrometry-based metabolomics provides an appropriate strategy for establishing such a framework, because it can describe broad chemical phenotypes and compare tissue-level metabolic variation [22,23]. Untargeted LC-MS/MS can be used to compare LC-MS-detectable non-volatile and low-volatility specialized features among plant organs, including flavonoid-, phenylpropanoid-, iridoid-, triterpenoid-, and lignan-related signals [24,25]. In the present study, this LC-MS/MS-based strategy was used to characterize chemical-feature patterns rather than to comprehensively profile volatile-oil composition. Reliable metabolomic comparison requires quality-control samples, feature filtering, appropriate data transformation, and statistical control of multiple testing [26,27,28,29]. Modern LC-MS workflows also depend on computational tools for peak detection, retention-time alignment, deconvolution, adduct or isotope grouping, and spectral-library matching, such as XCMS, CAMERA, and MS-DIAL [30,31,32,33]. These methods make it possible to compare organ-level chemical-feature patterns and to prioritize putative metabolite families for subsequent targeted verification [30,31,32,33,34,35].

In the present study, flowers, stems, and leaves of *V. negundo* were analyzed using an organ-resolved untargeted LC-MS/MS strategy. The objectives were to characterize global LC-MS feature profiles, compare organ-associated metabolic patterns and prioritize interpretable putative metabolite families related to plant specialized metabolism. This study provides an organ-resolved chemical-feature framework that connects the multi-part medicinal-use background of *V. negundo* with comparative metabolomic evidence from three aerial organs. The resulting putative family-level patterns may support the interpretation of existing medicinal-part use, guide the evaluation of potential resource organs, and provide targets for future authentic-standard confirmation, targeted quantification, bioactivity evaluation, and quality-marker development.

## 2. Materials and Methods

### 2.1. Plant Materials and Paired Sampling Design

Fresh flower, stem, and leaf tissues of *V. negundo* were collected on 14 July 2024 from Bozhou, Anhui Province, China (33.844457° N, 115.768112° E). The plant material was taxonomically authenticated by Chief Pharmacist Xiangsong Meng at Bozhou University. Voucher material (No. BZXY-20240714-01) was deposited in the cold-storage facility of Bozhou University.

Flower, stem, and leaf tissues were collected from the same three individual plants, forming *n* = 3 plant-individual-level paired biological blocks. In the source dataset, H denotes flower, J denotes stem, and Y denotes leaf. The paired structure was Plant 1: HJ-D-H1, HJ-D-J1, and HJ-D-Y1; Plant 2: HJ-D-H2, HJ-D-J2, and HJ-D-Y2; and Plant 3: HJ-D-H3, HJ-D-J3, and HJ-D-Y3. This organ-resolved paired design was used to reduce plant-individual background variation when comparing chemical-feature patterns among aerial organs. Because only three independent plants were available from this field collection, the dataset was used for exploratory paired organ-level analysis and was not intended to represent broad geographic, seasonal, or developmental variation in *V. negundo*. For stem sampling, approximately 1 cm segments were excised from the middle portion of second-year stems immediately below the current-year tender shoots. Thus, H, J, and Y identify flower, stem, and leaf tissue, respectively, whereas the terminal numerals 1–3 identify the plant individual; samples sharing the same numeral constitute one paired biological block.

The original photograph of the sampled flower-, stem-, and leaf-bearing material is provided in Figure S1.

### 2.2. Metabolite Extraction

Fresh samples (100 mg per sample) were ground in liquid nitrogen and extracted with 400 µL of precooled methanol/acetonitrile/water (2:2:1, *v*/*v*/*v*). The mixture was vortexed for 30 s, ultrasonicated for 20 min in a 4 °C water bath, incubated at −20 °C for 30 min and centrifuged at 14,000 rcf at 4 °C for 20 min. The extracts were vacuum-dried, reconstituted in 100 µL acetonitrile/water (1:1, *v*/*v*), vortexed, centrifuged and filtered through a 0.22 µm membrane before LC-MS/MS analysis. The methanol/acetonitrile/water mixture was selected as a broad untargeted extraction solvent to recover LC-MS-detectable metabolites across a relatively wide polarity range while promoting protein precipitation; it was not intended to target a single compound class.

### 2.3. LC-MS/MS Conditions

Chromatographic separation was performed by ultra-high-performance liquid chromatography (UHPLC) on an Agilent 1290 Infinity LC system (Agilent Technologies, Santa Clara, CA, USA) using a Waters ACQUITY UPLC BEH C18 column (1.7 µm, 2.1 mm × 100 mm; Waters Corporation, Milford, MA, USA). The column temperature was 40 °C, the flow rate was 0.4 mL/min, the injection volume was 2 µL, and the autosampler temperature was 4 °C. Mobile phase A was water containing 25 mM ammonium acetate and 0.5% formic acid, and mobile phase B was methanol. The gradient was 0–0.5 min, 5% B; 0.5–10.0 min, 5–100% B; 10.0–12.0 min, 100% B; 12.0–12.1 min, 100–5% B; and 12.1–16.0 min, 5% B.

Samples were injected in randomized order. Pooled quality-control (QC) samples were prepared by mixing equal aliquots of all study samples and were inserted before, during, and after the analytical sequence to monitor UHPLC-MS/MS stability. Solvent blank samples were checked before sample acquisition. Representative total ion chromatograms (TICs) and base peak chromatograms (BPCs) are provided in Figures S2–S4 as supplementary QC and signal-coverage evidence only. The LC-MS/MS strategy was used to compare LC-MS-detectable non-volatile and low-volatility features rather than to comprehensively characterize volatile-oil composition.

Mass-spectrometric acquisition was performed on an AB SCIEX TripleTOF 6600 instrument (SCIEX, Framingham, MA, USA) in positive and negative electrospray ionization modes. Source settings were Gas1 60, Gas2 60, curtain gas 30, a source temperature of 600 °C, and an ion spray voltage floating of +5500 V in positive mode and −5500 V in negative mode. The time-of-flight mass spectrometry (TOF MS) scan range was *m*/*z* 60–1000 and the product-ion scan range was *m*/*z* 25–1000. Information-dependent acquisition was performed in high-sensitivity mode with a declustering potential of ±60 V, a collision energy of 35 ± 15 eV, isotope exclusion within 4 Da, and ten candidate ions per cycle. Chromatograms presented in Supplementary Information are intended for QC verification and signal-coverage documentation only and were not used to judge differential LC-MS features.

### 2.4. Data Source, Filtering, and Normalization

The reanalysis used the vendor-processed MS-DIAL-aligned feature table rather than independent raw-data peak picking. The source files were generated in SCIEX .wiff/.wiff.scan format. According to the service-provider response, data processing was performed in MS-DIAL version 4.60 for peak detection, deconvolution, retention-time correction, peak alignment, peak-area extraction, and putative annotation, and ProteoWizard 3.0.6428 was listed in the processing information. The delivered dataset used for this manuscript was the vendor-processed aligned table. Key reported MS-DIAL parameters included an MS1 tolerance of 0.01, an MS2 tolerance of 0.025, a minimum peak height of 800, a minimum peak width of 3, and an alignment retention-time tolerance of 0.15.

The processed workbook contained feature IDs, retention time, *m*/*z*, adduct-related fields where available, formulae, putative compound names, superclass/class/subclass annotations, KEGG Compound IDs when available, vendor scores, biological sample intensities, and pooled-QC intensities. A vendor-reported quality-control relative standard deviation (QC RSD) column was present in the positive-ion sheet, whereas the negative-ion sheet did not contain an RSD field. To apply one consistent criterion across ion modes, pooled-QC RSD was independently recalculated from QC-1, QC-2, and QC-3 for every feature as the sample standard deviation divided by the mean and multiplied by 100%. Downstream QC filtering, paired organ-level statistics, visualization, and putative family-level summarization were performed independently in the present reanalysis.

Features were retained if they were detected in at least two of three samples in one or more organ groups and had pooled-QC relative standard deviation (RSD) of ≤30%. Although solvent blanks were tested before injection, no blank-channel data were provided by the analysis service, so background subtraction could not be performed on the feature matrix. Representative TICs and BPCs are provided in Figures S2–S4 as chromatographic quality-control and signal-coverage evidence only, not as independent evidence for organ-associated differential features. Retained feature intensities were sample-wise median-normalized and log2(x + 1)-transformed before PCA, correlation analysis, paired testing, and heatmap visualization. For sample-wise median normalization, each retained intensity was divided by the median of the nonzero retained-feature intensities in that sample and multiplied by the median of the 12 sample-specific medians (nine biological samples plus three pooled-QC samples), followed by log2(x + 1) transformation.

### 2.5. Exploratory Paired Feature-Level Screening

A plant-individual-level paired *t*-test was used as the primary exploratory feature-level screening method, with each plant serving as its own block across flower, stem, and leaf samples. Benjamini−Hochberg false discovery rate (FDR) correction was applied within each pairwise comparison. Features were classified as exploratory paired-screening features when |mean paired log2 difference| ≥ 1 and FDR < 0.05. These entries are reported as differential LC-MS features or putatively annotated features, not as confirmed metabolite identities. Statistical analysis was performed in Python 3.13.5. Numerical operations and median normalization used NumPy 2.3.5; paired *t*-tests used scipy.stats.ttest_rel in SciPy 1.17.0; and Benjamini−Hochberg correction used statsmodels.stats.multitest.multipletests (method = “fdr_bh”) in statsmodels 0.14.6.

Unsupervised principal component analysis (PCA) was used to evaluate global sample structure after QC filtering, median normalization, and log2 transformation. Because the dataset contained only three plant-individual blocks, supervised classification models such as partial least squares-discriminant analysis (PLS-DA), or orthogonal partial least squares-discriminant analysis (OPLS-DA) were not used as primary evidence. In small untargeted metabolomics datasets, such supervised models are susceptible to overfitting unless supported by adequate sample size and independent validation. Therefore, paired feature-level testing with Benjamini−Hochberg FDR correction was retained as the main exploratory screening strategy. PCA was implemented using sklearn.decomposition.PCA in scikit-learn 1.8.0 on the QC-filtered, median-normalized, and log2(x + 1)-transformed sample-by-feature matrix. The PCA implementation mean-centered each feature, with no additional unit-variance scaling. Figures were generated with Matplotlib 3.10.8.

### 2.6. Annotation and KEGG Reporting Boundary

Compound names were treated as vendor-labelled putative annotations. The service provider reported that annotations were generated from an in-house database using accurate mass, MS/MS spectra, adduct information, formula prediction, and database score. The vendor score was defined as an MS/MS similarity-based database-matching score from MS-DIAL ranging from 0 to 1. In the delivered workbook, the 1101 named database matches had scores from 0.700 to 0.976, and 1088 remained after QC filtering. These values were treated as vendor-derived database-matching scores and were not equated with a formal Schymanski confidence level. All compound names therefore remain putative annotations unless independently confirmed by orthogonal evidence [34,35,36].

No compounds were confirmed with authentic standards, and all reported labels therefore correspond to putative annotations. Chemical interpretation was restricted to family-level hypotheses unless authentic standards and reviewed MS/MS spectra became available. No quantitative or semi-quantitative comparison of individual putative compounds was performed; only inter-tissue LC-MS feature abundance trends were discussed at the metabolic-family level. Kyoto Encyclopedia of Genes and Genomes (KEGG) information was exported only as Compound ID context and was not used for pathway enrichment [34,35,36].

## 3. Results

### 3.1. Plant Materials and Analytical Workflow

Flower, stem, and leaf tissues from the same three *V. negundo* individuals were used to generate an organ-resolved paired LC-MS/MS dataset. This design allowed each plant to serve as its own biological block and provided a direct framework for comparing LC-MS-detectable chemical-feature patterns among aerial organs. The original photograph of the sampled flower-, stem-, and leaf-bearing material is provided in Figure S1. Table 1 summarizes the paired design and QC-filtering overview.

### 3.2. QC Filtering and Global Organ Structure

The source workbook contained 27,328 positive-ion and 16,229 negative-ion features. After pooled-QC RSD and missingness filtering, 26,317 positive-ion and 15,808 negative-ion features were retained, giving 42,125 retained LC-MS features for downstream paired organ-level screening. Across all detected features, 96.7% met the criterion of a QC RSD of ≤30%, supporting analytical repeatability for downstream exploratory analysis. Additional QC-filtering details are provided in Figure S5.

**Figure 1:**
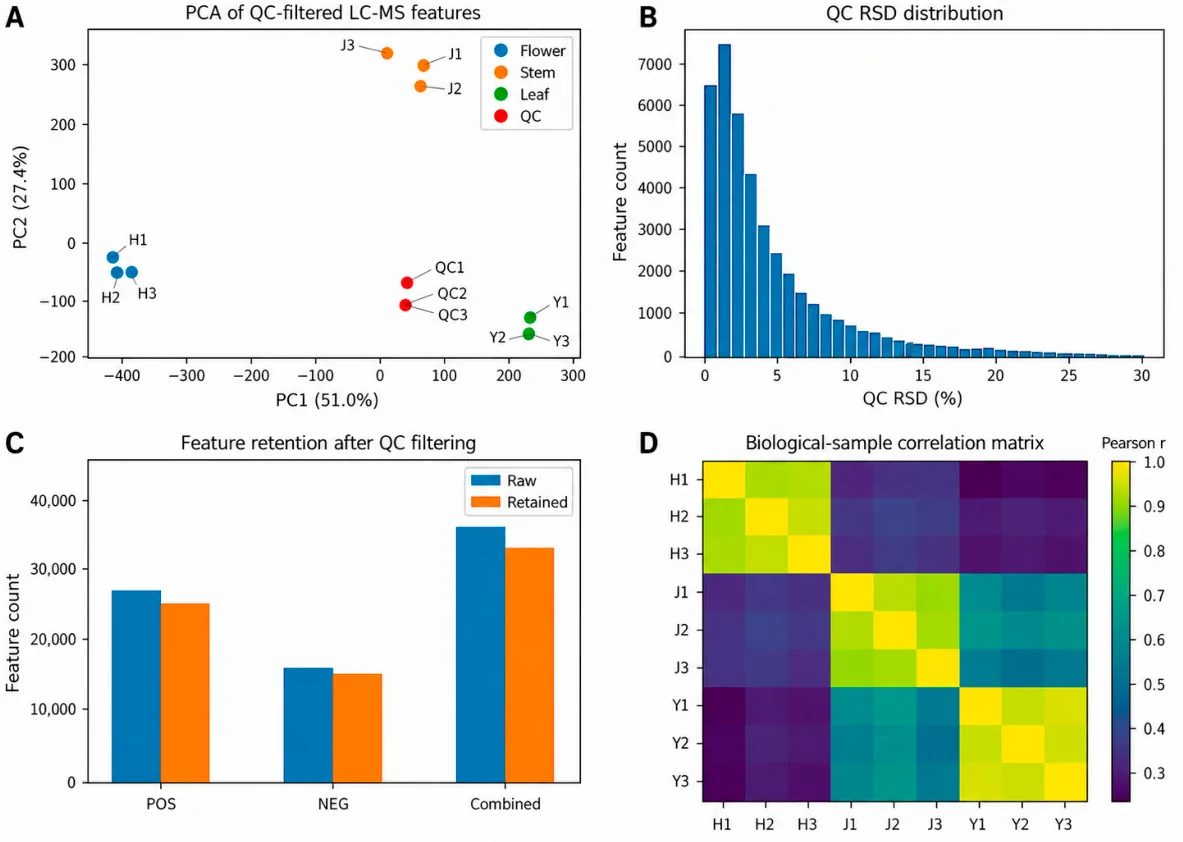
Quality control and global metabolic profiles after QC filtering, median normalization, and log2(x + 1) transformation. (**A**) principal component analysis (PCA) score plot of biological and pooled quality-control (QC) samples; principal component 1 (PC1) and principal component 2 (PC2) explain 51.0% and 27.4% of the variance, respectively. (**B**) QC relative standard deviation (RSD) distribution of retained features. (**C**) Feature retention after QC and missingness filtering. (**D**) Biological-sample correlation matrix. PCA was used as an unsupervised overview rather than as a supervised classification model. LC-MS, liquid chromatography–mass spectrometry.

PCA based on the retained, median-normalized, and log2(x + 1)-transformed feature matrix showed organ-associated separation among flowers, stems, and leaves, while QC samples clustered together. Under the fully specified reproducible Python workflow, principal component 1 (PC1) and principal component 2 (PC2) explained 51.0% and 27.4% of the variance, respectively. Pearson correlation analysis of QC samples, Hotelling’s T2 inspection, multivariate control charts, and QC RSD behavior in the analytical-service report further supported instrumental stability and repeatability. QC-TIC overlays in positive and negative ion modes and representative organ-level BPCs document chromatographic stability and signal coverage (Figures S2–S4). These chromatograms were used only as chromatographic quality-control and signal-coverage evidence and not as independent evidence for differential LC-MS features. Although the paired samples showed clear organ-associated separation in this dataset, this pattern should be interpreted within the limits of the present sampling site and paired plant blocks. The corresponding QC and global sample-structure results are summarized in Figure 1.

### 3.3. Annotation Landscape

**Figure 2:**
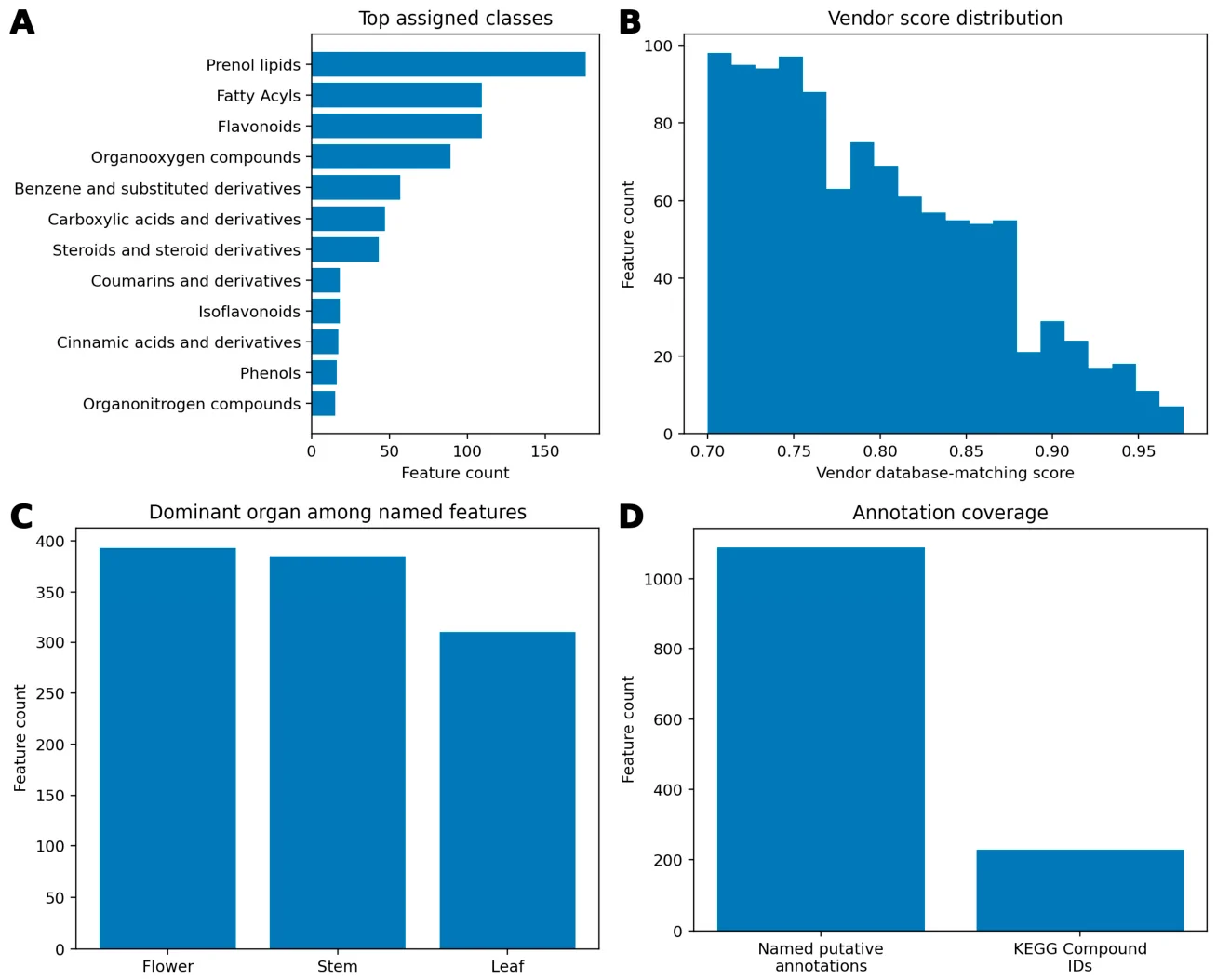
Annotation landscape of the retained LC-MS dataset. (**A**) Top assigned classes among named putative annotations with vendor scores of ≥0.7. (**B**) Distribution of vendor database-matching scores. (**C**) Dominant organ among higher-scoring named features. (**D**) Overall annotation coverage. Vendor scores are database-matching scores and do not confirm compound identities.

Among the 1088 QC-retained named putative annotations, frequently assigned feature classes included prenol lipids, fatty acyls, flavonoids, organooxygen compounds, benzenoid-related classes, carboxylic acids and derivatives, steroid-related features, isoflavonoids, coumarins, and cinnamic-acid derivatives. Features lacking interpretable superclass/class information were not interpreted as chemically defined classes in the main text. This annotation landscape indicates that the LC-MS dataset contained multiple feature families relevant to *V. negundo* chemistry and plant specialized metabolism, but the labels remain putative annotations rather than confirmed structures. The most frequently assigned classes are summarized in Table 2, and the overall annotation landscape is shown in Figure 2.

### 3.4. Exploratory Paired Differential Feature Screening

**Figure 3:**
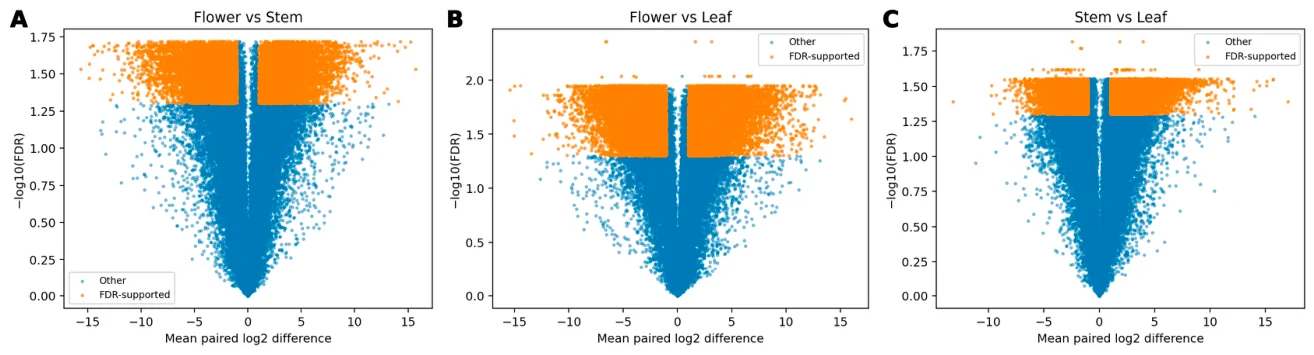
Exploratory paired feature-level screening across organs. (**A**) Flower vs. stem. (**B**) Flower vs. leaf. (**C**) Stem vs. leaf. Orange points represent paired FDR-supported features meeting the criteria of FDR < 0.05 and |mean paired log2 difference| ≥ 1. These plots are used for exploratory feature prioritization and do not indicate confirmed metabolite identities.

Using plant identity as the pairing factor, exploratory paired feature-level screening identified organ-associated differences. At the threshold of FDR < 0.05 and |mean paired log2 difference| ≥ 1, flower vs. stem, flower vs. leaf, and stem vs. leaf comparisons yielded 13,043, 22,836, and 13,393 paired-screening LC-MS features, respectively. These counts describe LC-MS features and do not represent unique metabolites because a single compound may generate multiple adducts, isotopes, in-source fragments, or redundant annotations. A supplementary summary distinguishing FDR-supported and nominal-only feature counts is provided in Figure S6. Directional feature counts are summarized in Table 3, and the corresponding paired feature-level screening is shown in Figure 3.

### 3.5. Overlap of Paired Screening-Supported Features

**Figure 4:**
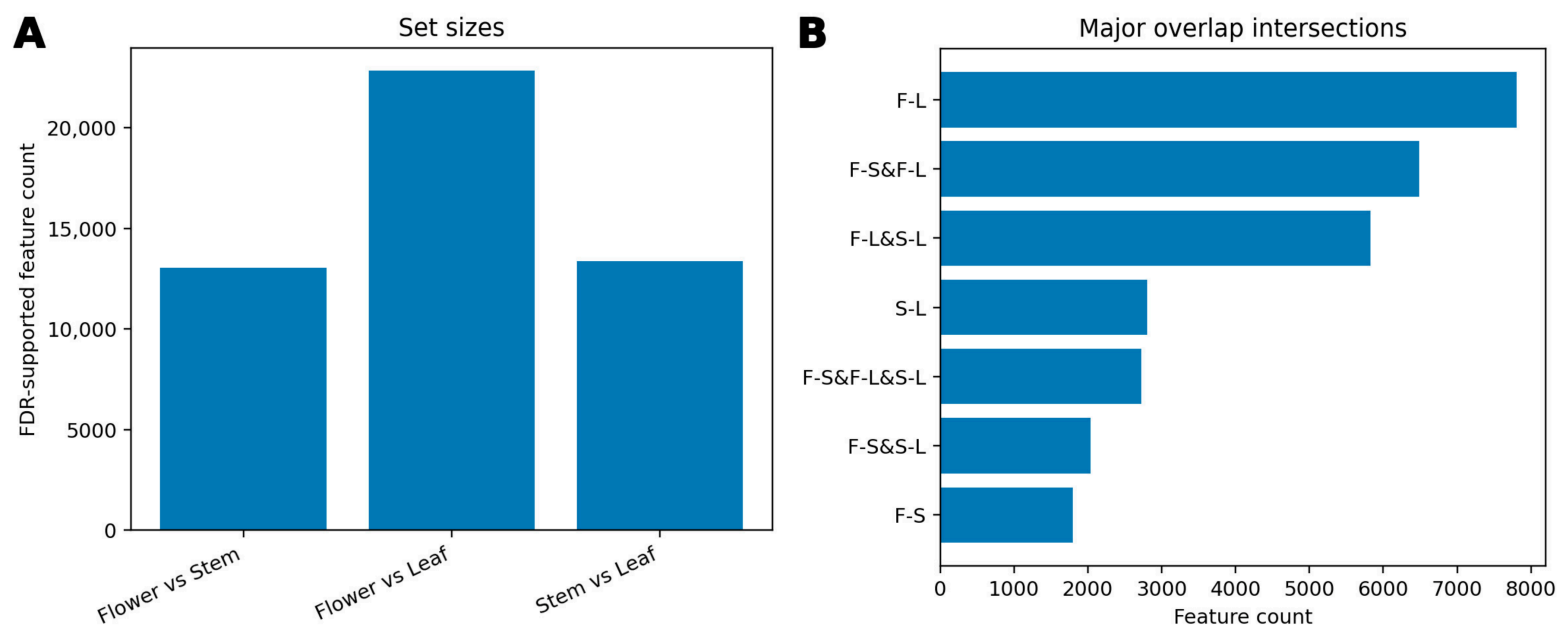
Overlap of paired FDR-supported features across the three organ comparisons. (**A**) Set sizes for paired screening-supported features. (**B**) Major overlap intersections among comparisons. Results were generated using FDR < 0.05 and |mean paired log2 difference| ≥ 1. Feature counts do not represent unique metabolite counts.

Overlap analysis separated broadly organ-associated features from comparison-specific signals. These paired screening-supported intersections are useful for prioritizing validation targets and for identifying chemical-feature groups that may distinguish one organ from the other two. However, the overlap structure is not equivalent to a nonredundant metabolite inventory because LC-MS feature redundancy, adduct formation, and putative annotation ambiguity remain present in the feature matrix. The major overlap patterns are summarized in Figure 4.

### 3.6. KEGG Compound ID Coverage

KEGG Compound IDs were available for only a limited subset of retained features. These IDs were used as contextual compound identifiers and putative family-level context only. They were not used for formal pathway enrichment or pathway-level interpretation because the dataset contains redundant LC-MS features and vendor-labelled putative annotations rather than nonredundant confirmed compounds. The corresponding KEGG Compound ID context is shown in Figure S7.

### 3.7. Organ-Associated Putative Family-Level Patterns

**Figure 5:**
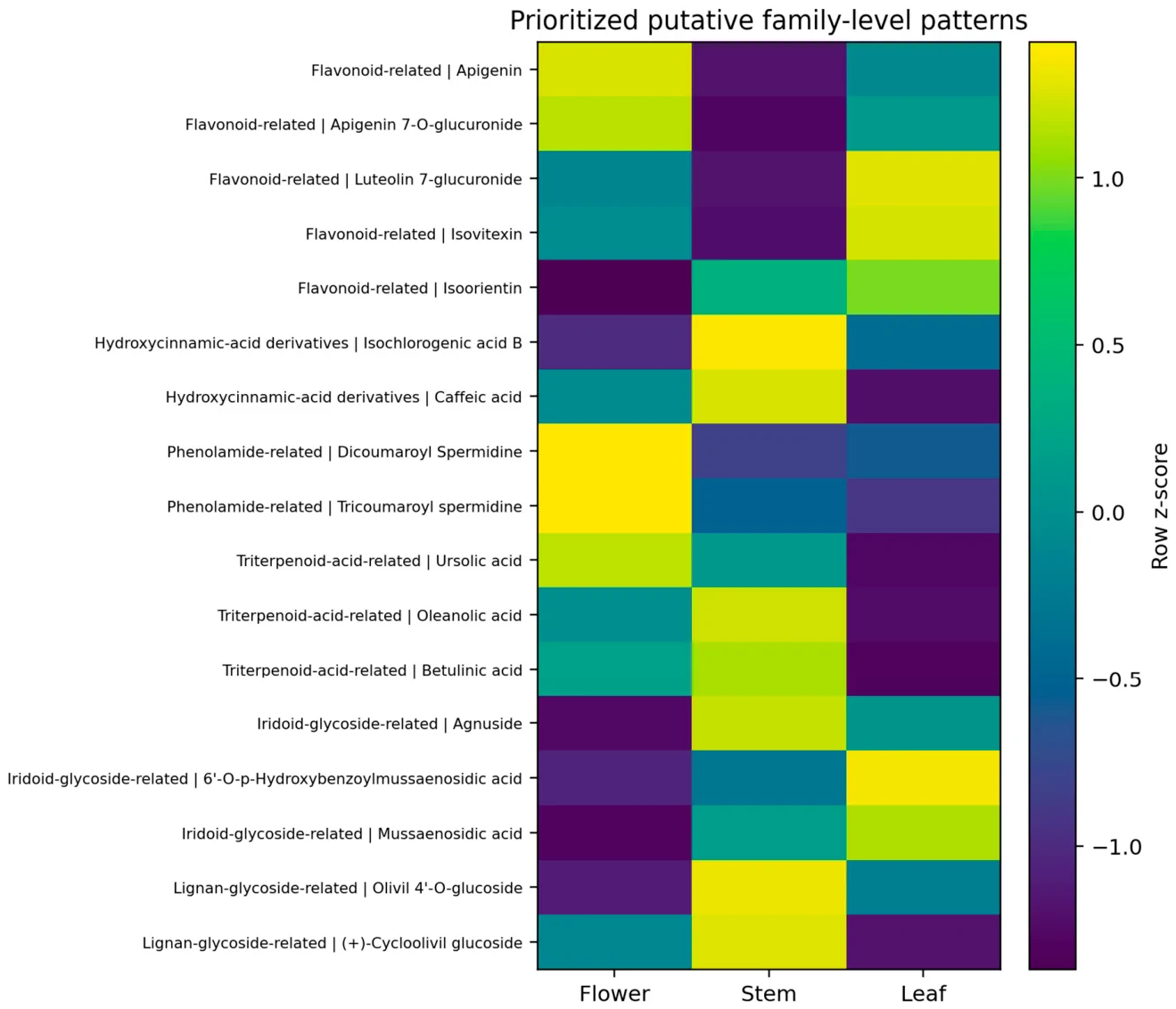
Condensed organ-associated putative family-level patterns in flower, stem, and leaf tissues. Heatmap values represent row-scaled z-scores calculated from group-mean log2-normalized intensities. The color scale indicates relative row z-scores, with higher and lower values shown according to the accompanying scale. Putatively annotated features were selected from higher-scoring vendor-labelled putative annotations with family-level relevance to Vitex chemistry or plant specialized metabolism. All compound names are putative annotations only and should not be interpreted as confirmed metabolite identities or validated diagnostic conclusions.

A validation-oriented set of higher-scoring named putative annotations was summarized at the family level to support subsequent targeted verification. These entries are presented as putative family-level interpretations rather than functionally or structurally validated metabolites. Drug, toxin, contaminant, or otherwise implausible database names were excluded from this descriptive shortlist. Because no authentic standards or manually reviewed MS/MS spectra were available in the reanalysis package, all entries are reported with explicit annotation-confidence boundaries. An expanded heatmap of selected putatively annotated features is provided in Figure S8. The condensed organ-associated putative family-level patterns are shown in Figure 5.

## 4. Discussion

This study provides a paired organ-resolved chemical-feature comparison of *V. negundo* aerial organs under a single untargeted LC-MS/MS workflow. The results showed that flowers, stems, and leaves could be separated at the global LC-MS feature level and that each organ contained different sets of paired screening-supported features. These findings support the rationale of moving *V. negundo* research beyond isolated single-organ extracts or individual compounds toward a comparative organ-level assessment. Because *V. negundo* has a multi-part medicinal and resource-use background, such organ-resolved information is useful for interpreting existing medicinal-part selection and for identifying underexplored aerial organs that merit subsequent targeted evaluation [1,2,20].

The putative family-level patterns provide a chemical basis for generating testable hypotheses rather than final conclusions about organ efficacy. Leaf-associated flavonoid-related putative signals, including luteolin-, vitexin/isovitexin-, and isoorientin-type annotations, are consistent with the role of leaves as photosynthetic and light-exposed tissues, where phenolic and flavonoid-related chemistry may contribute to photoprotection, redox regulation, and defense [15,16]. This interpretation is also compatible with previous *V. negundo* studies in which leaf materials or leaf extracts were frequently investigated for anti-inflammatory, analgesic, and antioxidant activities [3,4,5]. These relationships do not prove that the detected leaf-associated features mediate the reported activities, but they provide a focused chemical basis for subsequent targeted validation of leaf-related family-level signals. This organ-level interpretation is also consistent with previous *V. negundo* studies reporting phenolic-, flavonoid-, and iridoid-related chemistry in leaf-containing materials [8,9].

Stem-associated hydroxycinnamic-acid, caffeoylquinic-acid, coumarin, and lignan-related putative signals are compatible with a phenylpropanoid context, because stems contain vascular and supporting tissues in which phenolic acids and related conjugates may be linked to cell-wall-associated metabolism, lignification, and defense [17,18,37]. The enrichment of these putative family-level signals in stems suggests that stem chemistry should not be dismissed solely because leaves have received greater pharmacological attention. Instead, stem-associated phenylpropanoid- and lignan-related signals can be used to design targeted confirmation and bioactivity-screening experiments for underexplored aerial tissues. In the present study, the stem samples were approximately 1 cm middle segments of second-year stems immediately below current-year tender shoots. This developmental context is biologically compatible with a more mature support/transport tissue in which lignification-related metabolism may be more prominent, although no stem function was experimentally tested here.

Flower-associated flavone-type, phenolamide-related, and selected triterpenoid- or iridoid-related signals suggest a distinct subset of putative specialized features in reproductive tissues. Floral tissues often accumulate specialized metabolites involved in pigmentation, volatile emission, reproductive protection, and biotic interactions, although volatile-oil chemistry is more appropriately assessed by gas chromatography–mass spectrometry (GC-MS)-based approaches [7,19,38,39]. The present LC-MS/MS dataset therefore complements, rather than replaces, volatile-component studies. It provides non-volatile and low-volatility putative family-level signals that may help guide future studies on flower chemistry, while avoiding the claim that floral volatile profiles were comprehensively characterized. These possible links to reproductive-tissue functions are interpretive hypotheses based on organ biology and relative LC-MS feature patterns; no flower-specific biological function was experimentally validated in this study.

The results also refine how potential resource organs should be considered. The study does not establish flowers or stems as new medicinal parts, because such a conclusion would require pharmacological activity, safety, quality-standard, and resource-evaluation evidence. Instead, it provides an organ-level putative family-level map that can guide follow-up work. This distinction is important for medicinal-resource evaluation and quality-marker development, where chemical features should be linked with source specificity, measurability, biological relevance, and validation evidence before being used for quality assessment [20,21]. Thus, the putative family-level patterns reported here are most appropriately treated as starting points for subsequent standard-based confirmation, targeted quantification, and activity-oriented evaluation.

The present workflow differs from many earlier *V. negundo* studies by comparing flowers, stems, and leaves under the same analytical framework. In addition, the paired plant-block design reduced part of the plant-to-plant background variation by collecting different organs from the same individuals. The workflow also separated LC-MS-detectable non-volatile and low-volatility specialized features from volatile-oil chemistry, which is better addressed by GC-MS-based approaches [38,39]. Therefore, the results should be interpreted as an LC-MS/MS feature-level profile of non-volatile and low-volatility organ-associated chemical features rather than as a complete chemical inventory of *V. negundo* organs.

Annotation confidence remains a central boundary of this dataset. The vendor-provided score was used only as a database-matching score and not as a formal Schymanski confidence level. No authentic standards, retention-time matches, or systematic manual MS/MS confirmation were available for the complete dataset, so individual names remain putative [34,35,36]. Accordingly, Table 4 reports putative family-level interpretations rather than structurally or functionally validated metabolites. For the phenolamide-related entry, the exact regioisomeric attachment positions of the coumaroyl substituents cannot be uniquely assigned from the available MS/MS evidence. KEGG Compound IDs were used only as database context and were not used for pathway enrichment.

Several limitations should be considered. First, only three independent plants were included, and paired sampling cannot fully compensate for limited biological replication. Second, all compound labels remain putative annotations because no authentic standards, retention-time matching, or manually reviewed MS/MS confirmation were available; isomeric structures, glycosylation positions, stereochemistry, and co-eluting features therefore cannot be distinguished [34,35,36]. Third, solvent blank samples were checked before acquisition, but blank columns were not included in the delivered processed workbook, so downstream blank-background subtraction could not be performed. Fourth, the study covered LC-MS-detectable non-volatile and low-volatility features only and did not characterize volatile oil components. Fifth, no in vitro or in vivo bioactivity assays were included. Sixth, KEGG information was used only as Compound ID context and not for pathway enrichment or pathway-level interpretation.

The putative family-level shortlist should therefore be used as a starting point for targeted validation. Follow-up work should expand sampling across plants, seasons, growth stages, and habitats; include authentic standards and retention-time comparison where available; review diagnostic fragments manually; perform calibration or semi-quantitative response assessment; and evaluate prioritized features in appropriate bioactivity models. Such work would help determine whether organ-associated putative family-level patterns contribute to the chemical rationale of existing medicinal-part use or to the evaluation of potential resource organs in *V. negundo* [20,21].

## 5. Conclusions

This organ-resolved untargeted LC-MS/MS study retained 42,125 QC-filtered LC-MS features from flowers, stems, and leaves of three paired *V. negundo* plant individuals. PCA and paired feature-level screening indicated organ-associated LC-MS feature patterns, with hydroxycinnamic-acid derivative and lignan-related putative signals mainly associated with stems, flavonoid-related signals more evident in leaves, and flavone-, phenolamide-, and triterpenoid-related putative signals observed in flowers. The dataset provides a conservative organ-level prioritized feature list for future structural confirmation, targeted quantification, bioactivity evaluation, and medicinal-resource assessment of *V. negundo* aerial organs.

Because this was a small-sample exploratory study, all compound labels remain putative annotations without authentic-standard confirmation. The results should not be interpreted as confirmed metabolite identities, validated diagnostic or quality-assessment conclusions, pathway-level evidence, or pharmacological mechanisms. Instead, they provide an evidence-based validation-prioritization framework for linking multi-part medicinal use with organ-level chemical differentiation and for designing subsequent validation studies.

## Figures and Tables

**Table 1:** Study design and QC-filtering overview. Retained features met the criterion of a pooled quality-control (QC) relative standard deviation (RSD) of ≤30%. Service-provider QC evidence included total ion chromatogram (TIC) overlap, principal component analysis (PCA) clustering, QC Pearson correlations of >0.9, Hotelling’s T2 within the 99% confidence region, multivariate control charts within ±3 standard deviations (SD), and no available blank columns.

Ion Mode	Raw Features	Retained Features	Removed Features	Retention Rate (%)
POS	27,328	26,317	1011	96.3
NEG	16,229	15,808	421	97.4
Combined	43,557	42,125	1432	96.7

**Table 2:** Most frequently assigned classes among higher-scoring named putative annotations. Vendor scores are database-matching scores and are not formal Schymanski confidence levels. Note: features lacking superclass/class assignment were not interpreted as defined chemical classes.

Superclass	Class	Feature Count	Annotation Status
Lipids and lipid-like molecules	Prenol lipids	176	Putative (vendor score ≥ 0.700)
Lipids and lipid-like molecules	Fatty Acyls	109	Putative (vendor score ≥ 0.700)
Phenylpropanoids and polyketides	Flavonoids	109	Putative (vendor score ≥ 0.700)
Organic oxygen compounds	Organooxygen compounds	89	Putative (vendor score ≥ 0.700)
Benzenoids	Benzene and substituted derivatives	57	Putative (vendor score ≥ 0.700)
Organic acids and derivatives	Carboxylic acids and derivatives	47	Putative (vendor score ≥ 0.700)
Lipids and lipid-like molecules	Steroids and steroid derivatives	43	Putative (vendor score ≥ 0.700)
Phenylpropanoids and polyketides	Isoflavonoids	18	Putative (vendor score ≥ 0.700)
Phenylpropanoids and polyketides	Coumarins and derivatives	18	Putative (vendor score ≥ 0.700)
Phenylpropanoids and polyketides	Cinnamic acids and derivatives	17	Putative (vendor score ≥ 0.700)
Benzenoids	Phenols	16	Putative (vendor score ≥ 0.700)
Organic nitrogen compounds	Organonitrogen compounds	15	Putative (vendor score ≥ 0.700)

**Table 3:** Exploratory paired differential feature counts by organ comparison and direction. Values are LC-MS feature counts, not unique metabolite counts. FDR, false discovery rate.

Comparison	Direction	Paired FDR-Significant Features	Nominal-Only Features
Flower vs. Stem	Flower higher	7126	3536
Flower vs. Stem	Stem higher	5917	3223
Flower vs. Leaf	Flower higher	12,485	1862
Flower vs. Leaf	Leaf higher	10,351	1463
Stem vs. Leaf	Stem higher	6918	3923
Stem vs. Leaf	Leaf higher	6475	3587

**Table 4:** Representative organ-associated putative family-level interpretations prioritized for targeted validation. Full feature-level details, including feature IDs, RT, *m*/*z*, vendor score, mean paired log2 difference, FDR, and KEGG identifiers where available, are provided in Table S3. All entries are putative annotations and require structural validation. RT, retention time; FDR, false discovery rate; KEGG, Kyoto Encyclopedia of Genes and Genomes.

Putative Family-Level Interpretation	Representative Vendor-Derived Putative Annotations	Main Associated Organ(s)	Evidence Boundary	Recommended Validation Step
Flavonoid-related	Apigenin; apigenin 7-O-glucuronide; luteolin; luteolin 7-glucuronide; isovitexin; isoorientin	Leaf; flower	Putative family assignment; glycoside positions/isomers unresolved	Standards + RT matching + targeted MS/MS
Hydroxycinnamic-acid derivatives	Isochlorogenic acid B; 3,5-/4,5-dicaffeoylquinic-acid-related features; caffeic acid	Stem	Caffeoylquinic-acid positional isomers unresolved	Standards + chromatographic isomer separation
Phenolamide-related	Dicoumaroyl- and tricoumaroyl-spermidine-related features	Flower	Exact coumaroyl attachment positions cannot be uniquely assigned from the available MS/MS evidence	Targeted MS/MS + reference compounds
Triterpenoid-acid-related	Ursolic-acid-, oleanolic-acid-, and betulinic-acid-related features	Flower; stem	Isomeric triterpenoid acids unresolved	Standards + RT matching
Iridoid-glycoside-related	Agnuside; 6′-O-p-hydroxybenzoylmussaenosidic-acid-related feature; mussaenosidic-acid-related feature	Leaf; stem	Putative glycoside assignment; exact structure unresolved	Targeted MS/MS + standards
Lignan-glycoside-related	Olivil 4′-O-glucoside-related feature; cycloolivil-glucoside-related feature	Stem	Putative lignan-glycoside assignment; positional identity unresolved	Standards or curated-library confirmation

## Data Availability

The data supporting the findings of this study are included in the article, Supplementary Materials, and the submitted figure/table source-data and reproducibility package. The package contains the QC-retained feature matrix, complete paired statistics, retained named putative annotations, KEGG Compound ID context, figure/table source data, and the Python analysis workflow. The raw LC-MS/MS data and associated metabolomics metadata generated and analyzed in this study are available in the MetaboLights repository under accession number MTBLS15653. The original SCIEX .wiff/.wiff.scan files for all biological and pooled-QC injections are retained by the authors. Additional processed data and supporting materials are available from the corresponding authors upon reasonable request.

## Supplementary Materials

The following supporting information can be downloaded at: https://www.mdpi.com/article/10.3390/metabo16090633/s1, Figure S1, Original photograph of flower-, stem- and leaf-bearing material of *Vitex negundo* used in this study. The image documents the sampled aerial organs and supports sample traceability only; it does not substitute for taxonomic authentication or voucher documentation; Figure S2, Representative total ion chromatogram (TIC) overlays in positive and negative ion modes. (A) QC positive-ion TIC; (B) QC negative-ion TIC; (C) all-sample positive-ion TIC; (D) all-sample negative-ion TIC. The figure documents chromatographic stability and signal coverage only; Figure S3, Representative positive-ion base peak chromatograms (BPCs) for (A) flower, (B) stem and (C) leaf samples. The figure supports signal-coverage documentation for LC-MS-detectable non-volatile and low-volatility features in positive-ion mode; Figure S4, Representative negative-ion base peak chromatograms (BPCs) for (A) flower, (B) stem and (C) leaf samples. The figure supports signal-coverage documentation for LC-MS-detectable non-volatile and low-volatility features in negative-ion mode; Figure S5, QC filtering details for positive- and negative-ion LC-MS features. (A) Raw and retained feature counts by ion mode; (B) pooled quality-control (QC) relative standard deviation (RSD) distribution calculated from QC-1, QC-2 and QC-3. Retained features met the detection and pooled-QC RSD <= 30% criteria. Feature counts represent LC-MS features rather than unique metabolites; Figure S6, Paired differential-threshold summary showing FDR-supported and nominal-only feature counts by organ comparison and direction. Values are LC-MS feature counts and should not be interpreted as counts of unique metabolites; Figure S7, KEGG Compound ID context for paired organ comparisons. (A) Flower vs. stem; (B) flower vs. leaf; (C) stem vs. leaf; (D) KEGG Compound ID coverage by class. KEGG IDs are reported only as database identifiers and were not used for formal pathway enrichment or pathway-level interpretation; Figure S8, Expanded replicate-level heatmap of selected higher-scoring putatively annotated features. Values are row-scaled z-scores calculated from log2-normalized intensities for H1-H3, J1-J3 and Y1-Y3. Features were selected deterministically for visualization using vendor score and organ-level contrast magnitude; compound names remain putative annotations; Table S1, Retained LCMS feature matrix; Table S2, Complete paired feature statistics; Table S3, QC retained named putative annotations; and Table S4, KEGG Compound ID context. Figures S2–S4 are included only as chromatographic quality-control and signal-coverage evidence.

## References

[1] Vishwanathan A.S., Basavaraju R. (2010). A review on *Vitex negundo* L.—A medicinally important plant. Eur. J. Biol. Sci..

[2] Zheng C.J., Li H.Q., Ren S.C., Xu C.L., Rahman K., Qin L.P., Sun Y.H. (2015). Phytochemical and pharmacological profile of *Vitex negundo*. Phytother. Res..

[3] Dharmasiri M.G., Jayakody J.R.A.C., Galhena G., Liyanage S.S.P., Ratnasooriya W.D. (2003). Anti-inflammatory and analgesic activities of mature fresh leaves of *Vitex negundo*. J. Ethnopharmacol..

[4] Gupta R.K., Tandon V.R. (2005). Antinociceptive activity of *Vitex negundo* Linn. leaf extract. Indian J. Physiol. Pharmacol..

[5] Tiwari O.P., Tripathi Y.B. (2007). Antioxidant properties of different fractions of *Vitex negundo* Linn. Food Chem..

[6] Perumal Samy R., Ignacimuthu S., Sen A. (1998). Screening of 34 Indian medicinal plants for antibacterial properties. J. Ethnopharmacol..

[7] Sathiamoorthy B., Gupta P., Kumar M., Chaturvedi A.K., Shukla P.K., Maurya R. (2007). New antifungal flavonoid glycoside from *Vitex negundo*. Bioorganic Med. Chem. Lett..

[8] Huang M., Zhang Y., Xu S., Xu W., Chu K., Xu W., Zhao H., Lu J. (2015). Identification and quantification of phenolic compounds in *Vitex negundo* L. var. *cannabifolia* (Siebold et Zucc.) Hand.-Mazz. using liquid chromatography combined with quadrupole time-of-flight and triple quadrupole mass spectrometers. J. Pharm. Biomed. Anal..

[9] Wu Q., Zheng J., Yu Y., Li Z., Li Y., Hu C., Zhou Y., Chen R. (2024). Analysis of Antioxidant Compounds in *Vitex negundo* Leaves Using Offline 2D-LC-ECD and LC-MS/MS. Molecules.

[10] Zhu S., Shen Y., Zhang Q. (2025). Comparison in chemical constituents and anti-inflammatory and analgesic effects of different medicinal parts from a *Vitex negundo* L. var. *cannabifolia*. Fitoterapia.

[11] Wink M. (2003). Evolution of secondary metabolites from an ecological and molecular phylogenetic perspective. Phytochemistry.

[12] Hartmann T. (2007). From waste products to ecochemicals: Fifty years research of plant secondary metabolism. Phytochemistry.

[13] Pichersky E., Lewinsohn E. (2011). Convergent evolution in plant specialized metabolism. Annu. Rev. Plant Biol..

[14] Weng J.K., Philippe R.N., Noel J.P. (2012). The rise of chemodiversity in plants. Science.

[15] Agati G., Azzarello E., Pollastri S., Tattini M. (2012). Flavonoids as antioxidants in plants: Location and functional significance. Plant Sci..

[16] Treutter D. (2006). Significance of flavonoids in plant resistance: A review. Environ. Chem. Lett..

[17] Vogt T. (2010). Phenylpropanoid biosynthesis. Mol. Plant.

[18] Fraser C.M., Chapple C. (2011). The phenylpropanoid pathway in Arabidopsis. Arab. Book.

[19] Schiestl F.P. (2015). Ecology and evolution of floral volatile-mediated information transfer in plants. New Phytol..

[20] Liu C.X., Cheng Y.Y., Guo D.A., Zhang T.J., Li Y.Z., Hou W.B., Huang L.Q., Xu H.Y. (2017). A new concept on quality marker for quality assessment and process control of Chinese medicines. Chin. Herb. Med..

[21] Yang W., Zhang Y., Wu W., Huang L., Guo D., Liu C. (2017). Approaches to establish Q-markers for the quality standards of traditional Chinese medicines. Acta Pharm. Sin. B.

[22] Fiehn O. (2002). Metabolomics-the link between genotypes and phenotypes. Plant Mol. Biol..

[23] Hall R.D. (2006). Plant metabolomics: From holistic hope, to hype, to hot topic. New Phytol..

[24] Patti G.J., Yanes O., Siuzdak G. (2012). Metabolomics: The apogee of the omics trilogy. Nat. Rev. Mol. Cell Biol..

[25] Sumner L.W., Amberg A., Barrett D., Beale M.H., Beger R., Daykin C.A., Fan T.W., Fiehn O., Goodacre R., Griffin J.L. (2007). Proposed minimum reporting standards for chemical analysis. Metabolomics.

[26] Dunn W.B., Broadhurst D., Begley P., Zelena E., Francis-McIntyre S., Anderson N., Brown M., Knowles J.D., Halsall A., Haselden J.N. (2011). Procedures for large-scale metabolic profiling of serum and plasma using gas chromatography and liquid chromatography coupled to mass spectrometry. Nat. Protoc..

[27] Broadhurst D., Goodacre R., Reinke S.N., Kuligowski J., Wilson I.D., Lewis M.R., Dunn W.B. (2018). Guidelines and considerations for the use of system suitability and quality control samples in mass spectrometry assays applied in untargeted clinical metabolomic studies. Metabolomics.

[28] van den Berg R.A., Hoefsloot H.C.J., Westerhuis J.A., Smilde A.K., van der Werf M.J. (2006). Centering, scaling, and transformations: Improving the biological information content of metabolomics data. BMC Genomics.

[29] Benjamini Y., Hochberg Y. (1995). Controlling the false discovery rate: A practical and powerful approach to multiple testing. J. R. Stat. Soc. Ser. B.

[30] Smith C.A., Want E.J., O’Maille G., Abagyan R., Siuzdak G. (2006). XCMS: Processing mass spectrometry data for metabolite profiling using nonlinear peak alignment, matching, and identification. Anal. Chem..

[31] Kuhl C., Tautenhahn R., Bottcher C., Larson T.R., Neumann S. (2012). CAMERA: An integrated strategy for compound spectra extraction and annotation of liquid chromatography/mass spectrometry data sets. Anal. Chem..

[32] Tsugawa H., Cajka T., Kind T., Ma Y., Higgins B., Ikeda K., Kanazawa M., VanderGheynst J., Fiehn O., Arita M. (2015). MS-DIAL: Data-independent MS/MS deconvolution for comprehensive metabolome analysis. Nat. Methods.

[33] Tsugawa H., Ikeda K., Takahashi M., Satoh A., Mori Y., Uchino H., Okahashi N., Yamada Y., Tada I., Bonini P. (2020). A lipidome atlas in MS-DIAL 4. Nat. Biotechnol..

[34] Schymanski E.L., Jeon J., Gulde R., Fenner K., Ruff M., Singer H.P., Hollender J. (2014). Identifying small molecules via high resolution mass spectrometry: Communicating confidence. Environ. Sci. Technol..

[35] Blazenovic I., Kind T., Ji J., Fiehn O. (2018). Software tools and approaches for compound identification of LC-MS/MS data in metabolomics. Metabolites.

[36] Creek D.J., Dunn W.B., Fiehn O., Griffin J.L., Hall R.D., Lei Z., Mistrik R., Neumann S., Schymanski E.L., Sumner L.W. (2014). Metabolite identification: Are you sure? And how do your peers gauge your confidence?. Metabolomics.

[37] Boerjan W., Ralph J., Baucher M. (2003). Lignin biosynthesis. Annu. Rev. Plant Biol..

[38] Dudareva N., Pichersky E., Gershenzon J. (2004). Biochemistry of plant volatiles. Plant Physiol..

[39] Lisec J., Schauer N., Kopka J., Willmitzer L., Fernie A.R. (2006). Gas chromatography mass spectrometry-based metabolite profiling in plants. Nat. Protoc..

