# Supplementary material for: Organ-Resolved Untargeted LC-MS/MS Profiling of Putative Metabolite Families in Aerial Organs of Vitex negundo L
Source: Metabolites. 2026 Aug 31;16(9):633. doi: 10.3390/metabo16090633 (PMC13609388; doi:10.3390/metabo16090633)
Supplement: Supplementary file 1 [file metabolites-16-00633-s001.zip › Supplementary_Information_Vitex_negundo_CLEAN.pdf]

Supplementary Materials

Organ-Resolved Untargeted LC-MS/MS Profiling of Putative Metabolite Families in Aerial Organs of *Vitex negundo* L.

These supplementary materials support the organ-resolved untargeted LC-MS/MS comparison of flower, stem and leaf tissues of *Vitex negundo*. The figures and tables document sample traceability, chromatographic QC, feature filtering, exploratory paired differential screening, KEGG Compound ID context and putative family-level visualization. All LC-MS feature counts are feature-level signals rather than unique metabolites, and compound labels remain putative annotations requiring orthogonal confirmation.

Supplementary Figures

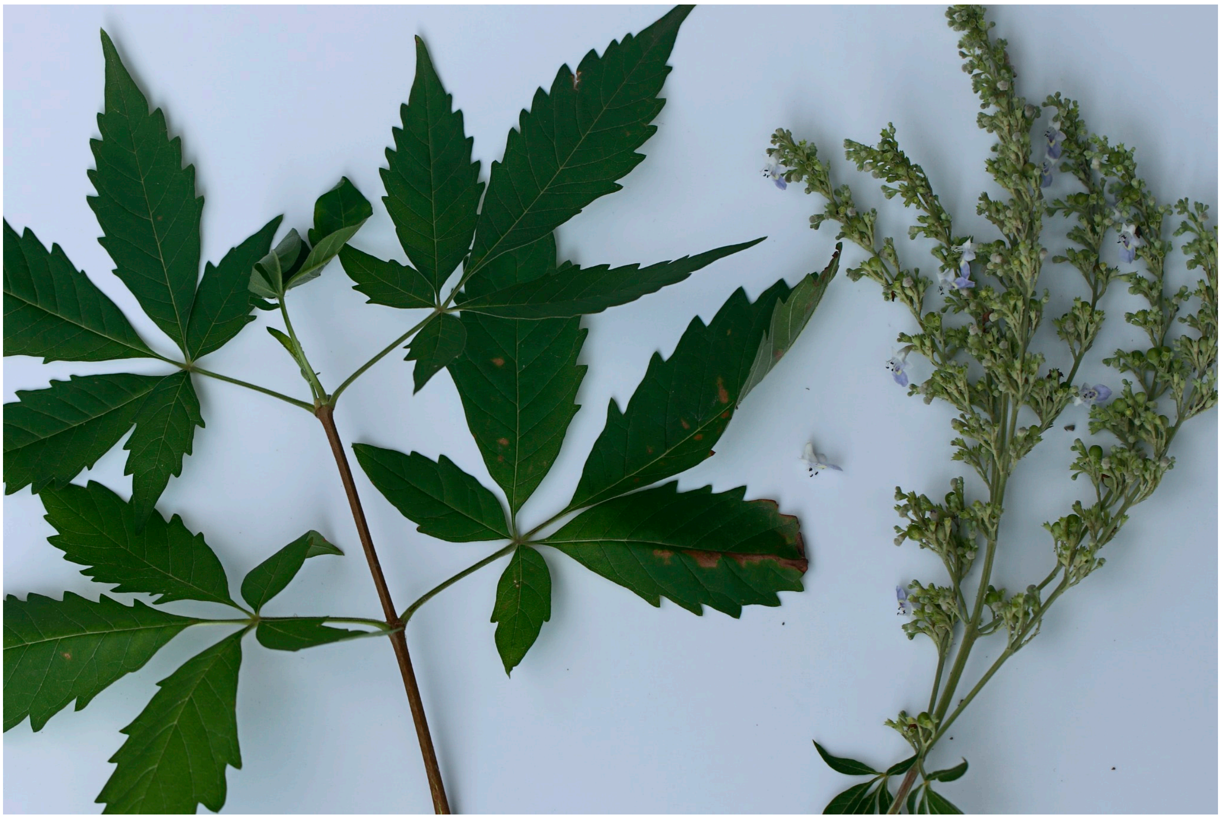

Figure S1. Original photograph of flower-, stem- and leaf-bearing material of *Vitex negundo* used in this study. The image documents the sampled aerial organs and supports sample traceability only; it does not substitute for taxonomic authentication or voucher documentation.

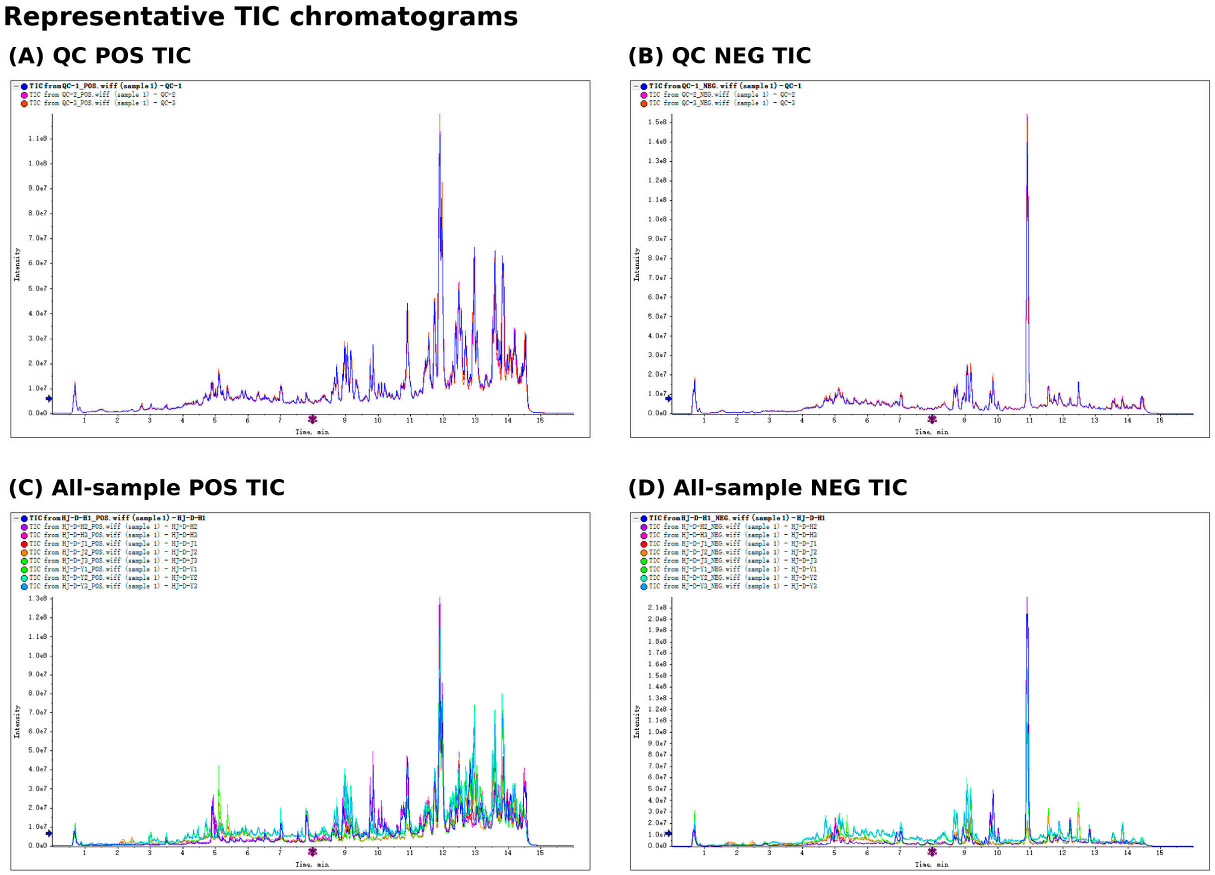

Figure S2. Representative total ion chromatogram (TIC) overlays in positive and negative ion modes. (A) QC positive-ion TIC; (B) QC negative-ion TIC; (C) all-sample positive-ion TIC; (D) all-sample negative-ion TIC. The figure documents chromatographic stability and signal coverage only.

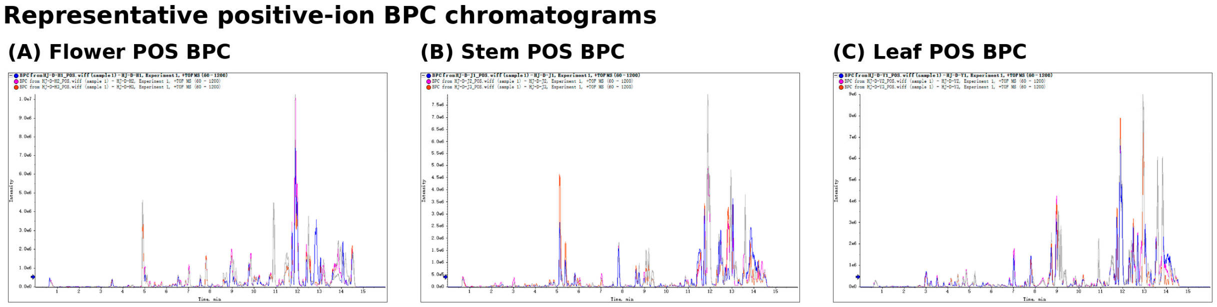

Figure S3. Representative positive-ion base peak chromatograms (BPCs) for (A) flower, (B) stem and (C) leaf samples. The figure supports signal-coverage documentation for LC-MS-detectable non-volatile and low-volatility features in positive-ion mode.

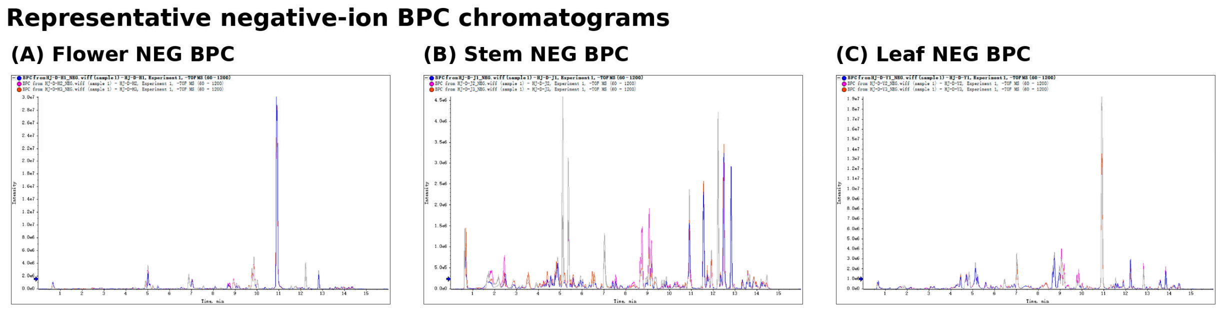

Figure S4. Representative negative-ion base peak chromatograms (BPCs) for (A) flower, (B) stem and (C) leaf samples. The figure supports signal-coverage documentation for LC-MS-detectable non-volatile and low-volatility features in negative-ion mode.

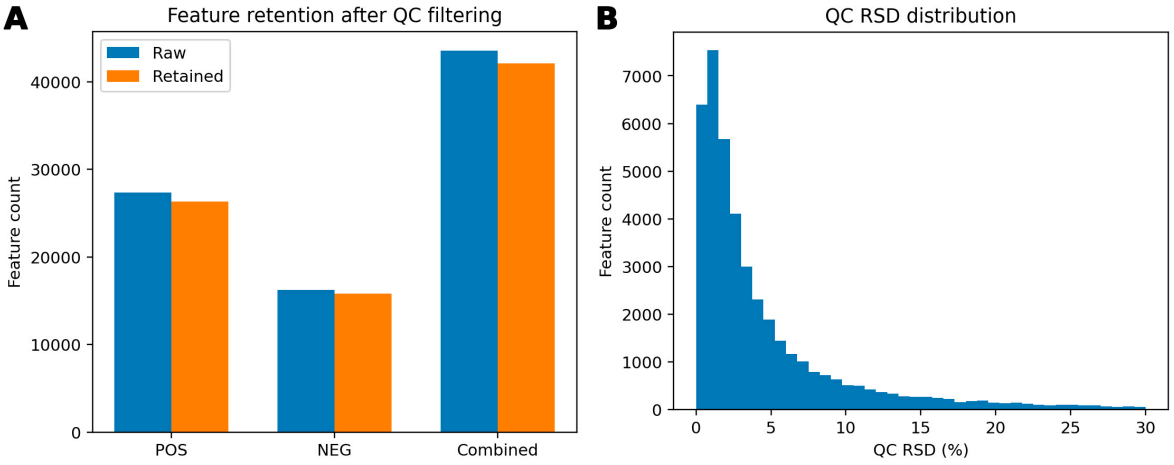

Figure S5. QC filtering details for positive- and negative-ion LC-MS features. (A) Raw and retained feature counts by ion mode; (B) pooled quality-control (QC) relative standard deviation (RSD) distribution calculated from QC-1, QC-2 and QC-3. Retained features met the detection and pooled-QC RSD <= 30% criteria. Feature counts represent LC-MS features rather than unique metabolites.

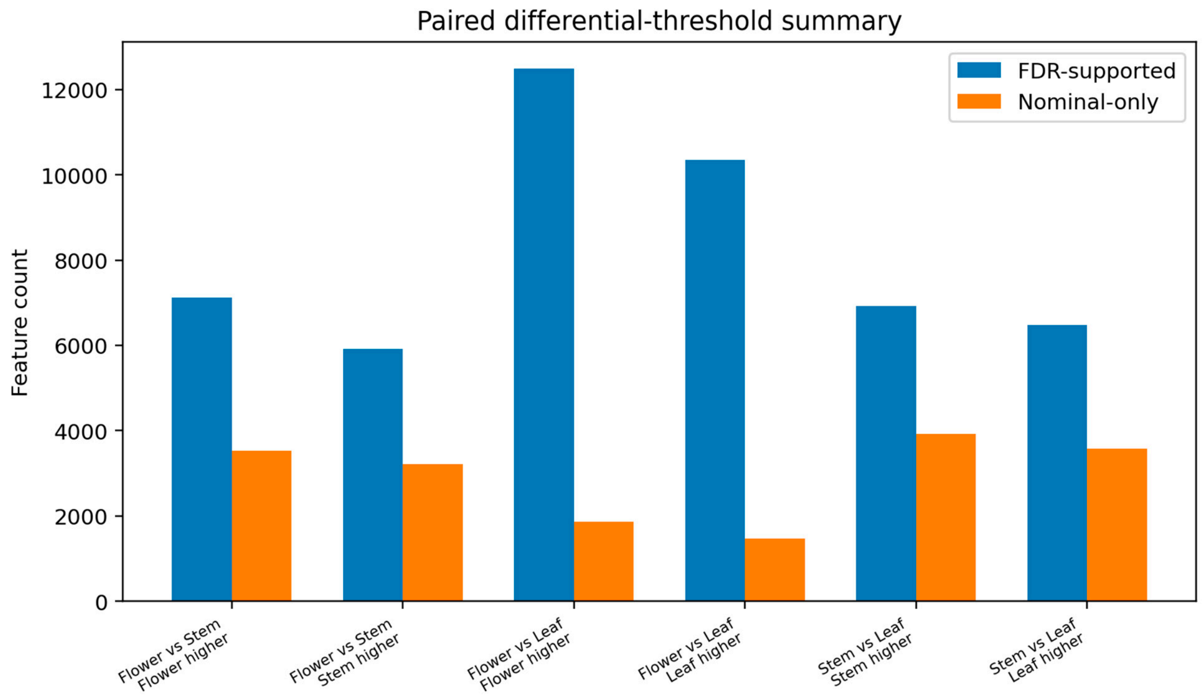

Figure S6. Paired differential-threshold summary showing FDR-supported and nominal-only feature counts by organ comparison and direction. Values are LC-MS feature counts and should not be interpreted as counts of unique metabolites.

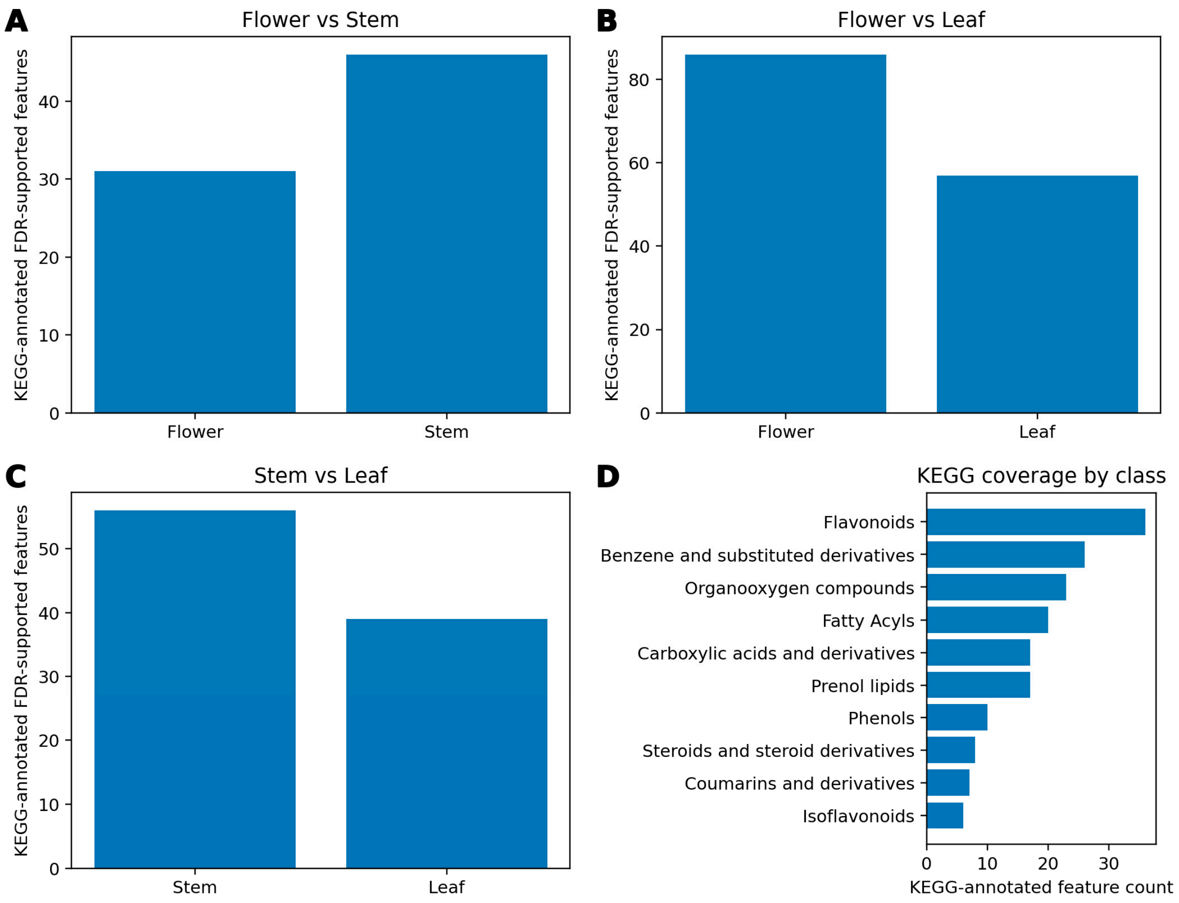

Figure S7. KEGG Compound ID context for paired organ comparisons. (A) Flower vs. stem; (B) flower vs. leaf; (C) stem vs. leaf; (D) KEGG Compound ID coverage by class. KEGG IDs are reported only as database identifiers and were not used for formal pathway enrichment or pathway-level interpretation.

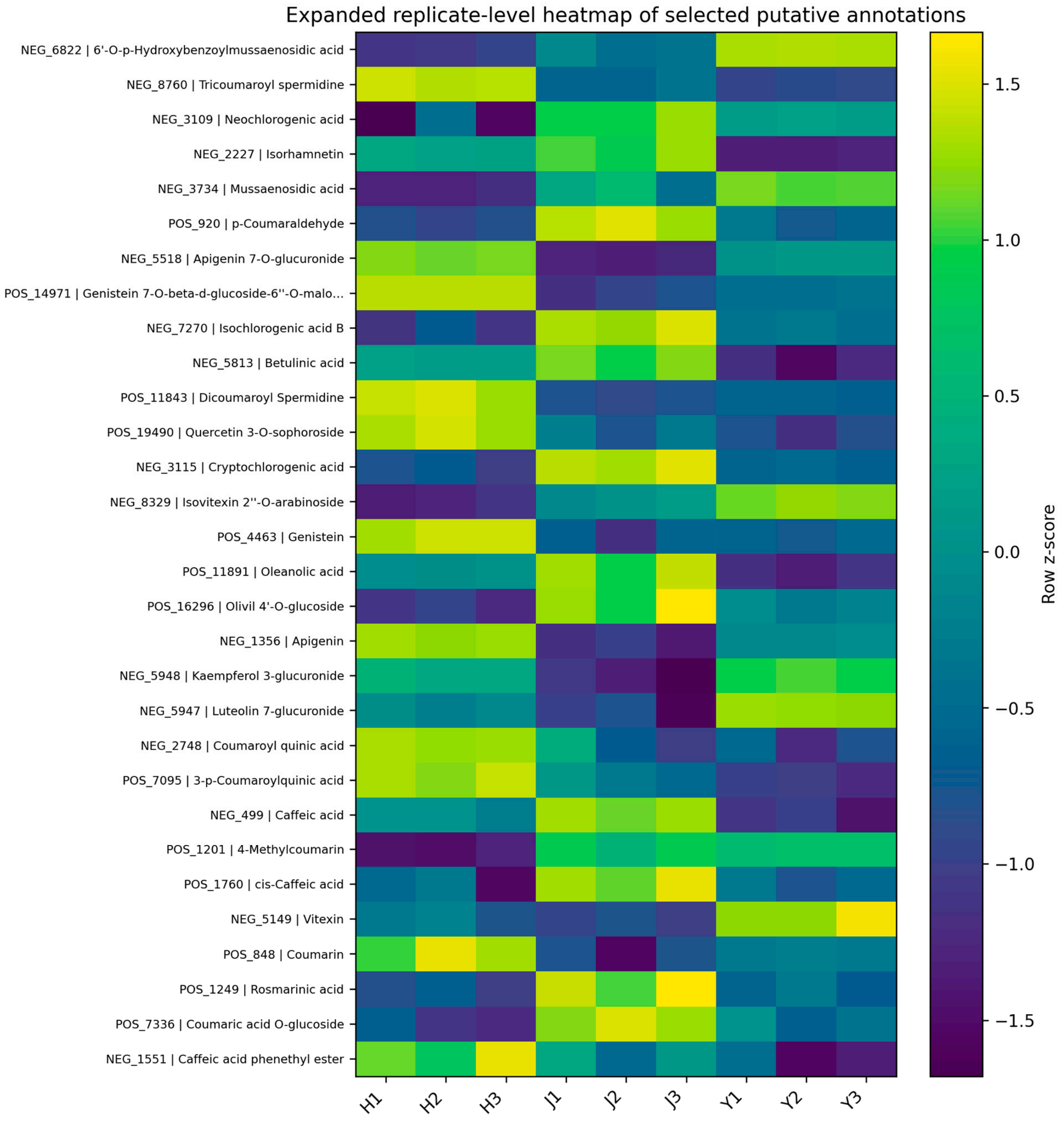

Figure S8. Expanded replicate-level heatmap of selected higher-scoring putatively annotated features. Values are row-scaled z-scores calculated from log2-normalized intensities for H1-H3, J1-J3 and Y1-Y3. Features were selected deterministically for visualization using vendor score and organ-level contrast magnitude; compound names remain putative annotations.

Supplementary Tables

Table	Content	Reporting boundary
Table S1	QC-retained LC-MS feature matrix after the detection and pooled-QC RSD filtering rules.	Feature intensities are LC-MS feature-level signals and are not unique metabolite abundances.
Table S2	Complete paired differential feature statistics for flower vs. stem, flower vs. leaf and stem vs. leaf comparisons.	FDR-supported and nominal-only entries are exploratory feature-level screening results.
Table S3	QC-retained named putative annotations with feature identifiers, vendor scores, organ-level abundance	All compound names remain putative annotations and require authentic-standard or curated-library

Table	Content	Reporting boundary
	summaries and validation-oriented statistics.	confirmation.
Table S4	KEGG Compound ID export and related database context.	KEGG entries are Compound IDs only and were not used for pathway enrichment, pathway regulation or metabolic flux interpretation.

Interpretation and Reproducibility Notes

- TIC and BPC chromatograms are provided as chromatographic quality-control and signal-coverage evidence only; they should not be interpreted as independent differential-feature, confirmed-metabolite, biomarker or bioactivity evidence.
- Feature-level counts are not equivalent to unique metabolite counts because one compound may generate multiple adducts, isotopes, in-source fragments or redundant annotations.
- The vendor score was defined as an MS/MS similarity-based database-matching score from MS-DIAL ranging from 0 to 1. Scores are used only to rank putative database matches and are not formal Schymanski confidence levels.
- A vendor-reported RSD column was present in the positive-ion sheet but not in the negative-ion sheet. For consistency, pooled-QC RSD was independently recalculated for both ion modes from QC-1, QC-2 and QC-3 using sample standard deviation divided by the mean and multiplied by 100%.
- Solvent blanks were checked before sample acquisition, but blank columns were not included in the delivered processed workbook; therefore, additional blank-background subtraction could not be performed in the downstream reanalysis.
- The paired design reduces part of the plant-individual background variation, but n = 3 plant blocks means paired p-values and FDR results remain exploratory screening evidence.
- KEGG entries are Compound IDs only and should not be interpreted as pathway enrichment, pathway association, pathway regulation, pathway activity or metabolic flux.
- PCA was reproduced in Python 3.13.5 using scikit-learn 1.8.0 after the stated median normalization and log2(x + 1) transformation; no additional unit-variance scaling was applied.
- Putative family-level panels are intended for targeted validation prioritization and should not be interpreted as confirmed metabolite identities, biomarkers, quality markers, validated medicinal-part evidence or pharmacological mechanisms.
- Numerical source data and scripts for the data-derived main and supplementary figures/tables are supplied in the figure/table source-data and reproducibility package. Figures S2-S4 are original vendor-exported TIC/BPC chromatograms derived from the original SCIEX acquisitions. The authors retain the corresponding .wiff/.wiff.scan files for all biological and pooled-QC injections in both ion modes; these raw files are available to editors/reviewers upon reasonable request during peer review and will be deposited in a third-party data repository upon acceptance, with the repository accession/identifier added to the final Data Availability Statement.
